# An exploratory Case Report: integrated genomic and transcriptomic profiling of a spontaneous facial tumor in the endangered Milu deer (*Elaphurus davidianus*)

**DOI:** 10.3389/fvets.2026.1933048

**Published:** 2026-10-22

**Authors:** Ying Zhang, Zhibin Cheng, Yunfang Shan, Qingxun Zhang, Pan Zhang, Jiade Bai, Qingyun Guo, Zhenyu Zhong

**Affiliations:** Beijing Milu Ecological Research Center, Beijing Academy of Science and Technology, Beijing, China

**Keywords:** case report, facial tumor, Milu deer, somatic mutations, transcriptomics, wildlife conservation

## Abstract

The Milu deer (*Elaphurus davidianus*), also known as Père David's deer, has experienced severe population bottlenecks. While infectious diseases are closely monitored, baseline data on sporadic non-communicable diseases, such as neoplasia, remain scarce for wildlife disease surveillance. To date, the molecular mechanisms driving spontaneous tumorigenesis in this species remain uncharacterized. This study provides the first integrated characterization of a single facial tumor in the Milu deer, combining histopathological analysis, whole-genome sequencing (WGS), and RNA sequencing (RNA-seq). Histopathological analysis confirmed that the tumor is an epithelial neoplasm exhibiting significant mesenchymal features, indicative of epithelial-mesenchymal transition (EMT). RNA-seq differential expression analysis identified 6,537 differentially expressed genes (DEGs), while WGS revealed 114 high-confidence, tumor-specific non-synonymous somatic mutations. Functional enrichment analysis demonstrated that oncogenesis in this specific specimen is potentially driven by dysregulated cell cycle, cytoskeletal reorganization, and metabolic reprogramming mediated by PI3K-Akt and mTOR signaling. Notably, the human papillomavirus (HPV) infection pathway emerged as significantly enriched among the DEGs. We emphasize that this enrichment does not provide evidence of viral involvement, as no direct viral detection was performed; rather, it exclusively reflects the severe dysregulation of highly conserved host cellular networks implicatedin cell cycle, apoptosis, and immune regulation. By integrating mutational and transcriptomic data, we prioritized ten putative tumor-associated genes, including *LGALS3, DMBT1, CHAF1A, GON4L*, and *SIRPA*, which are linked to metastasis, immunosuppression, and proliferation. This case study provides the first integrated molecular profile of a facial tumor in the Milu deer, offering critical insights for health management of this endangered species and establishing a novel framework for comparative oncology that will inform future clinical diagnoses of neoplasms in this population.

## Introduction

The Milu deer, an iconic species that was previously extinct in the wild, stands as a hallmark of successful captive breeding and reintroduction in conservation history (1). However, this recovery has been accompanied by a profound genetic bottleneck, raising concerns about the population's susceptibility to diseases, including potential neoplastic conditions (2). While research has traditionally focused on their population genetics and behavioral ecology (3–5), the health of individual Milu deer, particularly concerning non-communicable diseases like neoplasia, warrants attention for comprehensive disease surveillance and animal welfare. Documenting the pathological and preliminary molecular features of spontaneous tumorigenesis in this species is therefore valuable for accumulating baseline veterinary data and informing captive health management.

Spontaneous facial tumors in wildlife represent a distinct and understudied category of neoplastic diseases with unique cancer biology. Unlike transmissible facial tumors such as devil facial tumor disease (DFTD) in Tasmanian devils, which spreads via direct transfer of living cancer cells between individuals (6, 7), these tumors can originate from diverse cell lineages, including epithelial, mesenchymal, or mixed epithelial-mesenchymal components, and exhibit a wide spectrum of biological behaviors, ranging from benign growths to locally invasive or metastatic malignancies (8). In domestic animals, well-documented spontaneous facial tumors include squamous cell carcinomas, mast cell tumors, and melanomas, with etiologies linked to chronic ultraviolet exposure, viral infections, or breed-specific genetic predispositions (9). In wild cervids, however, the molecular etiology and pathological mechanisms of spontaneous facial tumors remain largely unexplored. While virus-induced cutaneous masses, particularly papillomavirus (PV)-associated fibropapillomas, are well-documented in several cervid species, including the roe deer papillomavirus (CcaPV1) endemic in European roe deer (*Capreolus capreolus)* (10) and the novel red deer papillomavirus (CePV1) characterized in *Cervus elaphus* (11), both of which closely resemble deer papillomavirus (DPV) infections in North American white-tailed deer, comprehensive genomic investigations of sporadic, non-viral neoplasms remain remarkably scarce. Although sporadic cases have been reported in related deer species (12), the lack of species-specific molecular tools and the difficulty of obtaining paired tumor-normal tissues from protected species have hindered comprehensive investigations. For the Milu deer, a species that has undergone a severe genetic bottleneck (13), elucidating the molecular underpinnings of spontaneous facial tumorigenesis is particularly critical, as reduced genetic diversity may profoundly influence tumor susceptibility and shape the mutational landscape that drives malignant transformation in this iconic species.

Carcinogenesis results from the progressive accumulation of genetic and transcriptomic alterations that disrupt cellular homeostasis and ultimately drive tumor development (14). Among these alterations, somatic mutations, especially single nucleotide variants (SNVs) that arise in tumor cells, constitute a major class of genomic changes underlying tumorigenesis (15). By contrast, germline single nucleotide polymorphisms (SNPs) are inherited variants present in all cells of an organism and occur frequently across the human genome at an average rate of approximately one per 1–2 kb (16). While somatic mutations primarily induce functional changes within tumor cells, germline SNPs serve as valuable biomarkers for investigating evolutionary dynamics and disease susceptibility. Somatic mutations can occur throughout the genome, for example, non-synonymous mutations in coding regions alter amino acid sequences, thereby potentially changing protein structure and function. Meanwhile, synonymous or non-coding mutations may influence gene expression, mRNA stability, or subcellular localization, all of which can contribute to malignant transformation (17).

The emergence of high-throughput sequencing technologies, particularly the integration of whole-genome sequencing (WGS) and RNA sequencing (RNA-seq), has significantly enhanced our ability to decipher complex genotype-phenotype relationships. This multi-omics integration enables a more robust screening of candidate genes for complex traits compared to single-method approaches (18, 19), markedly accelerating the identification of functional mutations and differentially expressed genes (DEGs) that underpin complex diseases like cancer (20–24). Applying these integrated omics approaches to non-model organisms with unique biological traits can reveal novel insights into conserved and species-specific disease mechanisms. The Milu deer provides a striking example, exhibiting a physiological paradox that makes it a compelling model for tumorigenesis research. Despite showing remarkable proliferative capacity during annual antler regeneration without neoplastic transformation (25). Cancer incidence rates are ~5 times lower in deer than in other mammals according to the records from both the Philadelphia (0.4%−0.8%) and San Diego (2.1%−4.6%) zoos (26). However, these figures are based solely on captive populations, and comparable data from wild populations are currently unavailable. Given the challenges in detecting and reporting neoplasms in free-ranging animals, it is likely that many cases in the wild remain undocumented. Therefore, the cited zoo-based cancer incidence rates may not accurately represent the true burden in wild Milu deer. Future comprehensive monitoring in natural habitats is needed to establish baseline neoplasia rates in wild populations. However, the genetic basis underlying tumor development in Milu deer remains unexplored, primarily due to the scarcity of available biological material from this protected species.

To address this gap, we conducted a case study integrating WGS and RNA-seq, complemented by detailed histopathological and immunohistochemical analyses of matched tumor and adjacent normal tissues from Milu deer. WGS enabled the identification of both germline SNPs and high-confidence tumor-specific somatic mutations, while RNA-seq quantified associated transcriptomic changes. Our primary objective was to identify candidate genes exhibiting both genetic alterations and transcriptional dysregulation, thereby pinpointing key molecular drivers of tumorigenesis. We expected the tumor to display classical malignancy hallmarks such as dysregulated cell cycle, cytoskeletal reorganization, and metabolic reprogramming, which would be reflected by characteristic histopathological and molecular features of an aggressive epithelial tumor undergoing epithelial-mesenchymal transition (EMT). This case study provides the first integrated genomic, transcriptomic, and histopathological profile of neoplasia in Milu deer, offering valuable insights to guide future molecular pathology and comparative oncology research.

## Materials and methods

### Experimental animals and ethical approval

Facial tumor and matched adjacent non-tumor tissues were collected from a deceased Milu deer at the Beijing Milu Ecological Research Center (Beijing, China). Specifically, a single, well-demarcated facial tumor mass (approximately 3.5 cm × 2.0 cm × 1.5 cm in size) was surgically excised. From the same anatomical region, a matched sample of macroscopically normal tissue was collected approximately 2.0 cm from the tumor margin to ensure the absence of visible neoplastic infiltration. All procedures were performed in accordance with standard ethical practices for the handling of animal specimens. Upon collection, samples were snap-frozen in liquid nitrogen and stored at −80 °C until nucleic acid extraction. A representative macroscopic photograph of the resected tumor and adjacent tissue is provided in Supplementary Figure S1.

### Histopathology and immunohistochemistry (IHC)

For histopathological evaluation, 5-μm-thick frozen sections were prepared from both the facial tumor and adjacent normal tissues. Sections were stained with Hematoxylin and Eosin (H&E) using standard protocols to evaluate tissue morphology. Immunohistochemical staining was also performed on frozen sections of the facial tumor and adjacent normal muscle tissue. Following methanol fixation, sections were subjected to antigen retrieval in Tris-EDTA buffer (pH 8.0) using a heat-mediated method. Endogenous peroxidase was blocked with 3% hydrogen peroxide. Sections were then incubated overnight at 4 °C with primary antibodies against Pan-cytokeratin (Pan-CK; 1:200; GB152053, Servicebio), Vimentin (1:200; GB15192, Servicebio), SOX10 (1:200; GB15782, Servicebio), S100 (1:500; GB15359, Servicebio), CD3 (1:500; GB151137, Servicebio), and CD68 (1:1000; GB153109, Servicebio). After washing, sections were incubated with HRP-conjugated secondary antibody (G1302, Servicebio) or (G1301, Servicebio), and signals were visualized using DAB chromogen. Sections were counterstained with hematoxylin, dehydrated, and mounted. Images were captured using a light microscope (E100, Nikon) and a digital slide scanner (LG-S80, Servicebio).

### Transcriptome sequencing (RNA-seq)

Frozen tissue samples were first pulverized into fine powder in liquid nitrogen using a chilled mortar and pestle to ensure thorough homogenization and to minimize RNA degradation. Total RNA was extracted with RNAiso Plus (Takara, 9109) and further purified using the HiPure Universal RNA Mini Kit (Magen, M028-00). During RNA extraction, the powdered tissue was immediately transferred to RNAiso Plus reagent and homogenized by vortexing following the manufacturer's instructions. RNA integrity was assessed with an Agilent 2100 Bioanalyzer, with RIN values of 8.5. RNA purity was determined by the 260/280 and 260/230 absorbance ratios measured on a NanoDrop spectrophotometer, with values of 1.95 and 2.05, respectively. Libraries were prepared with the NEBNext Ultra II RNA Library Prep Kit and sequenced on an Illumina NovaSeq X Plus (2 × 150 bp). Raw sequencing reads were processed for quality control and adapter removal using fastp (version 0.23.4) with the following parameters: bases with Phred quality scores below 20 were trimmed from both ends of the reads, and reads shorter than 50 nucleotides after trimming were discarded. Adapter sequences were automatically detected and removed. The quality of the sequencing data before and after preprocessing was assessed using FastQC (version 0.12.1) with default parameters. After adapter trimming and QC, reads were aligned to the red deer (*Cervus elaphus*) reference genome (Assembly: GCF_019320065.1) using HISAT2 (v2.0.5) with default parameters, due to the unavailability of a completed genome assembly for Milu deer. A comprehensive summary of the sequencing quality control metrics, including clean read counts, Q20/Q30 scores, error rate, mapping rates, GC content, adapter contamination, and insert size, is provided in Supplementary Table S2. Raw RNA-seq data are available in the Genome Sequence Archive (GSA) under CRA032811.

### Quantitative real-time PCR (qRT-PCR)

Total RNA was extracted from the excised facial tumor and matched adjacent non-tumor tissues using TRIzol reagent (Invitrogen, 15596026CN). cDNA was synthesized from 1 μg of total RNA with oligo (dT) primers and PrimeScript RT kit (Toyobo, FSQ-301) following the manufacturer's instructions. qPCR was conducted using SYBR Green I Master Mix (Vazyme, Q412-02), with GAPDH serving as the control. Cycling conditions consisted of an initial denaturation at 95 °C for 5 min, followed by 40 cycles of 95 °C for 10 s and 60 °C for 30 s. Relative mRNA expression levels were calculated using the 2^−ΔΔCt^ method, normalized to the respective control group. Primer sequences are detailed in Supplementary Table S1.

### Differential expression analysis

Gene-level counts were obtained with featureCounts (v1.5.0-p3). Given the absence of biological replicates, differential expression analysis was performed using the NOISeq R package (version 2.38.0), a non-parametric statistical method specifically designed for single-sample or low-replicate RNA-seq data. Gene expression was normalized using the RPKM method within NOISeq to account for differences in sequencing depth and gene length. DEGs were identified based on the computed probability of differential expression (prob). Genes with a prob value ≥0.9 were considered statistically significant DEGs. DEGs with a positive M-value (log_2_ fold change) were classified as upregulated in the tumor tissue, and those with a negative M-value as downregulated.

### Differential alternative splicing analysis

Differential alternative splicing (DAS) events between tumor and adjacent non-tumor tissues were analyzed using rMATS (v4.2.0) with default parameters. The software identifies five types of alternative splicing events: skipped exon (SE), alternative 5' splice site (A5SS), alternative 3' splice site (A3SS), mutually exclusive exons (MXE), and retained intron (RI). DAS events were considered significant with a false discovery rate (FDR) < 0.05.

### Functional enrichment analysis

GO and KEGG enrichment analyses for the identified DEGs were conducted using the clusterProfiler R package (v3.8.1). Terms and pathways with an adjusted *p*-value < 0.05 were considered statistically significant.

### Whole-genome resequencing (WGS)

Genomic DNA from tumor and adjacent non-tumor samples underwent WGS. Libraries were prepared by Novogene Biotech with fragmentation (Covaris), end repair, A-tailing, adapter ligation, size selection, and PCR amplification. Insert size distributions were assessed by AATI fragment analyzer, and libraries quantified by qPCR. Qualified libraries were sequenced on a DNBSEQ-T7 (MGI; 2 × 150 bp). Reads were trimmed with fastp (v0.20.0) and aligned to the reference genome (GCF_019320065.1) using BWA-MEM (v0.7.17), with duplicates removed by SAMtools. The average sequencing depth was 26.41 × for the control group and 54.95 × for the tumor group. Variant calling was performed using GATK (v4.1.1.0) following the best practices for somatic mutation detection in paired tumor-normal samples. Variants were filtered with the following criteria: MQ < 40.0, QD < 2.0, FS > 30.0, DP < 10, and QUAL < 20. Annotation was performed using snpEff (v4.3.1q). All WGS data are deposited under CRA033238.

### Somatic mutation detection and analysis

Since a fully assembled and annotated reference genome for Milu deer is not currently available, we aligned sequencing reads to the closely related red deer genome. While this approach introduces potential risks of misinterpreting fixed species-level differences as somatic mutations, this bias was systematically mitigated by our matched tumor-normal paired sequencing design. Because fixed inter-species SNPs are present in both the tumor and the adjacent normal tissue of the same individual, the GATK (v4.1.1.0). Mutect2 pipeline effectively identifies these shared variants as non-somatic background and filters them out. To further ensure high confidence, we implemented a conservative, multi-tiered filtering strategy.

Somatic mutations were identified using GATK (v4.1.1.0). Mutect2 in tumor-normal paired mode. To prioritize high-confidence, tumor-specific non-synonymous somatic mutations, we applied additional filters: (1) the somatic mutation site was present exclusively in tumor cells and absent in the matched normal control tissue (thereby excluding germline SNPs and fixed species-level divergences relative to the red deer reference). (2) variants were required to be located within coding sequences (CDS) and cause non-synonymous changes; (3) minimum alternative allele depth in the tumor sample ≥10; and (4) tumor variant allele frequency (VAF) >0.10, a standard threshold utilized in bulk tumor sequencing to confidently exclude low-frequency sequencing artifacts while capturing subclonal tumor heterogeneity (27, 28).

### Integrated analysis to prioritize putative tumor-associated genes

Somatic mutation calling: high-confidence, tumor-specific nonsynonymous somatic mutations were identified from WGS data using SAMtools with stringent filters (see Section 2.9). Expression intersection: the list of mutated genes was then intersected with the list of DEGs identified from RNA-seq analysis (defined as prob value ≥0.9). A gene was considered a high-priority candidate only if it harbored a tumor-specific nonsynonymous mutation and was significantly differentially expressed in the tumor compared to matched normal tissue. This conservative, two-tiered filtering strategy was designed to identify genes that are both genetically altered and transcriptionally dysregulated, generating hypotheses for their potential functional involvement in tumorigenesis.

## Results

## Histopathological and immunohistochemical characterization of the spontaneous facial tumor

To establish the biological nature and classification of the spontaneous facial tumor in the Milu deer, we first performed H&E staining. The adjacent normal tissue showed regular skeletal muscle fibers (Figure 1A'), whereas the tumor tissue exhibited malignant features including disorganized cell nests, adenoid structures, and significant nuclear pleomorphism, indicating an infiltrative epithelial neoplasm (Figure 1A). Further characterization by IHC showed robust cytoplasmic positivity for Pan-CK within the tumor nests (Figure 1B), and widespread Vimentin expression (Figure 1C), suggesting an epithelial-derived tumor with mesenchymal features. Crucially, the tumor cells were completely negative for SOX10 (Figure 1D) and S100 (Figure 1E). The absence of these markers, consistent with the results in adjacent muscle (Figures 1D', E'), definitively excludes a neural crest origin such as melanoma or schwannoma.

**Figure 1:**
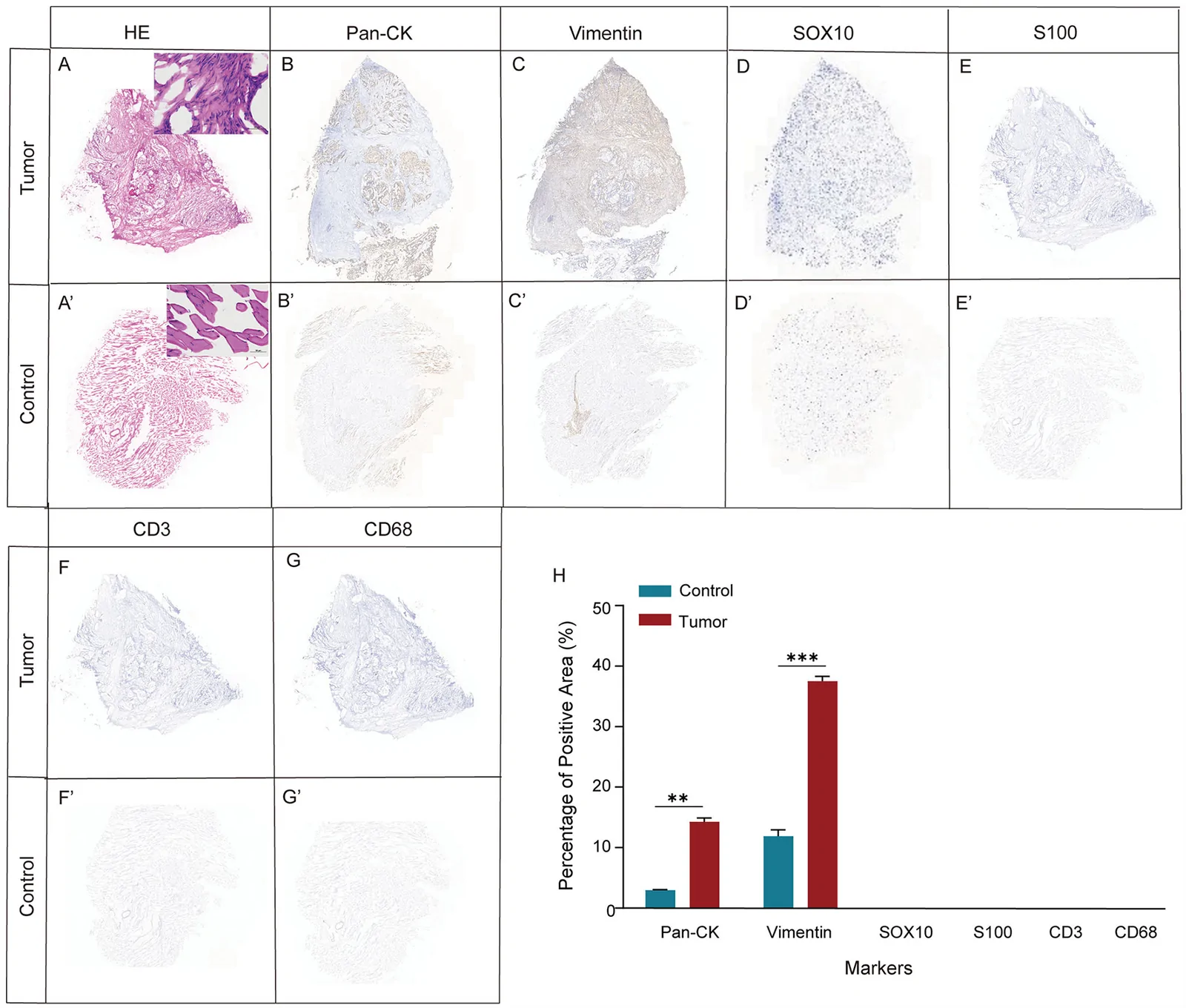
Histopathological and Immunohistochemical characterization of the spontaneous facial tumor and adjacent normal muscle tissue in the Milu deer. **(A, A')** show H&E staining; insets in **(A, A')** present higher-magnification views (400 × ). Panels **(B, B')**, **(C, C')**, **(D, D')**, **(E, E')**, **(F, F')**, and **(G, G')** show immunostaining for Pan-CK, Vimentin, SOX10, S100, CD3, and CD68, respectively. **(H)** Quantitative analysis of the percentage of positive area for each marker in tumor and adjacent normal tissues is shown. Means and SEMs (*n* = 3; Student's *t* test). **p < 0.01, ***p < 0.001.

Furthermore, to evaluate the host immune response within the tumor microenvironment, we examined the presence of tumor-infiltrating immune cells. IHC staining, showed that the tumor was completely negative for CD3 (T cell marker) and CD68 (macrophage marker; Figures 1F–G). Quantitative analysis of the IHC signals was conducted (Figure 1H). These findings, combined with the positive staining for Pan-CK and Vimentin, characterize the lesion as an “immune-cold” epithelial tumor with significant mesenchymal features.

## RNA-Seq reveals divergent gene expression profiles between tumor and adjacent non-tumor tissues

RNA-seq was performed to reveal the gene expression pattern between tumor and adjacent non-tumor tissues. Due to the rarity of the clinical sample, the analysis was conducted on a single tumor and a matched control, and thus the results represent individual observational trends. RNA-seq data quality was confirmed by high Q20/Q30 scores and mapping rates (Supplementary Table S2). Among 11,680 expressed genes, 7,399 were co-expressed across tissues (Figure 2A). DEGs between tumor and adjacent tissues were shown as a hierarchical clustering map (Figure 2B). A total of 6,537 DEGs were identified between tumor and adjacent tissues, comprising 5,423 upregulated and 1,114 downregulated genes, which collectively serve to clearly distinguish the tumor samples from the non-tumor counterparts (Figure 2C). All DEGs are presented in Supplementary Table S3. Additionally, some differentially alternative splicing (DAS) events were also detected, and skipped exon was a major part of DAS (Figure 2D). To validate the RNA-seq results, qPCR was performed on a subset of key candidate genes including *CHAF1A, LGALS3, DMBT1*, and *COG2*. The qPCR data showed expression trends consistent with RNA-seq findings, which confirm the reliability of the transcriptomic profiling (Figure 2E).

**Figure 2:**
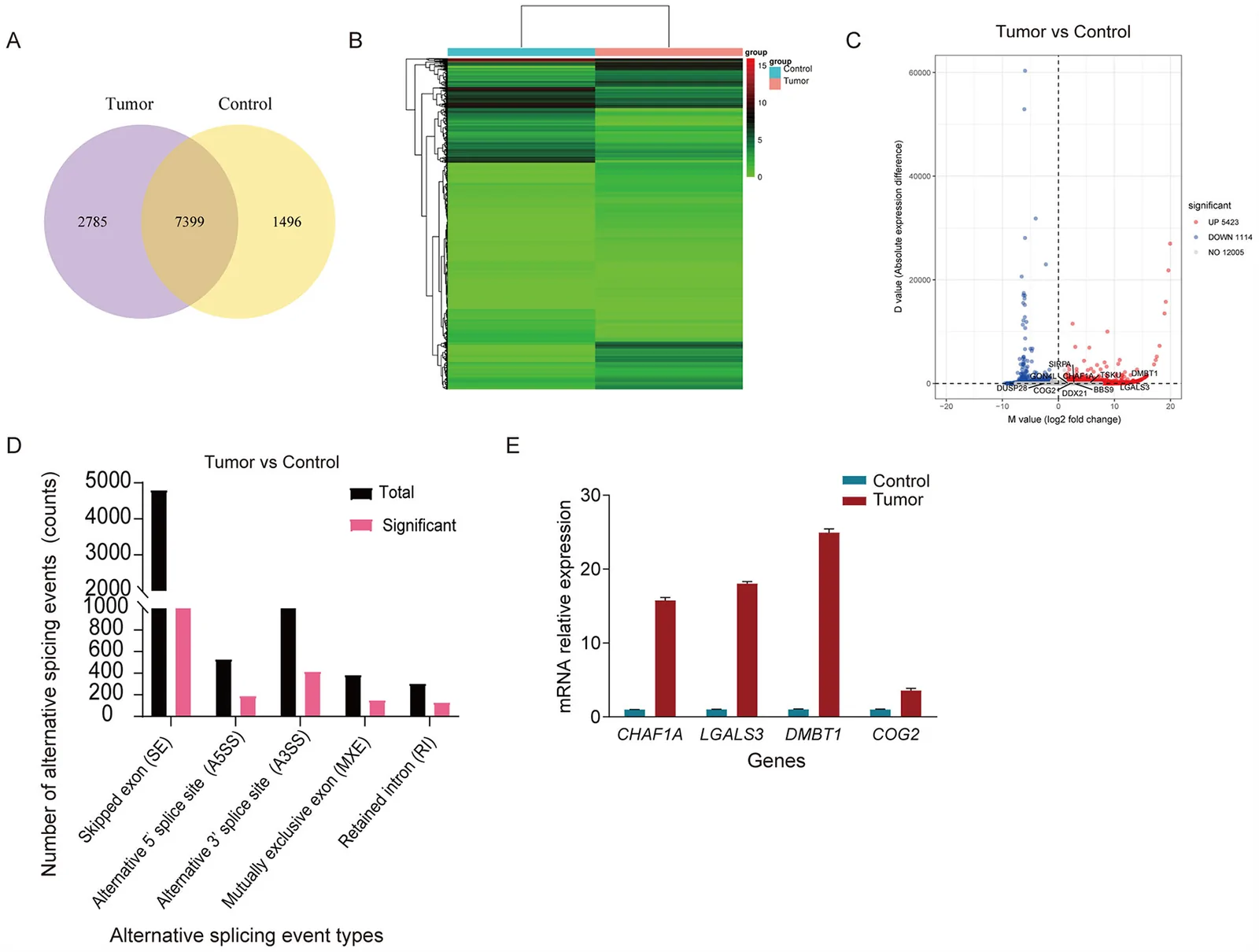
Transcriptome sequencing analysis of tumor and adjacent non-tumor tissues. **(A)** Venn diagram showing the number of genes expressed in tumor and adjacent non-tumor tissues. **(B)** The hierarchical clustering map of DEGs between tumor and adjacent non-tumor tissues. In the heatmap, red indicates higher expression levels, while green indicates lower expression levels relative to the mean. **(C)** Volcano plot of all DEGs, with the M value (log2 fold change) on the x-axis and the D value (expression difference) on the y-axis. Red dots denote significantly upregulated genes, blue dots denote significantly downregulated genes, and gray dots represent non-significant genes. **(D)** The number of different alternative splicing events identified from the RNA-seq data. **(E)** qRT-PCR analysis of *CHAF1A, LGALS3, DMBT1*, and *COG2* in control and tumor tissue.

## Functional enrichment analysis suggests potential metabolic reprogramming and signaling dysregulation in tumor pathogenesis

To infer the potential the functional landscape underlying tumorigenesis, we performed GO and KEGG enrichment analyses on the 6,537 DEGs. In the molecular function category, the most prominently enriched terms were related to dysregulated protein kinase activity and small GTPase binding. Consistent with these findings, biological process analysis indicated a trend toward enrichment of protein phosphorylation and small GTPase-mediated signal transduction. In the cellular component category, DEGs were significantly enriched in cytoskeletal structures such as actin cytoskeleton, indicating active cytoskeletal reorganization (Figure 3A).

**Figure 3:**
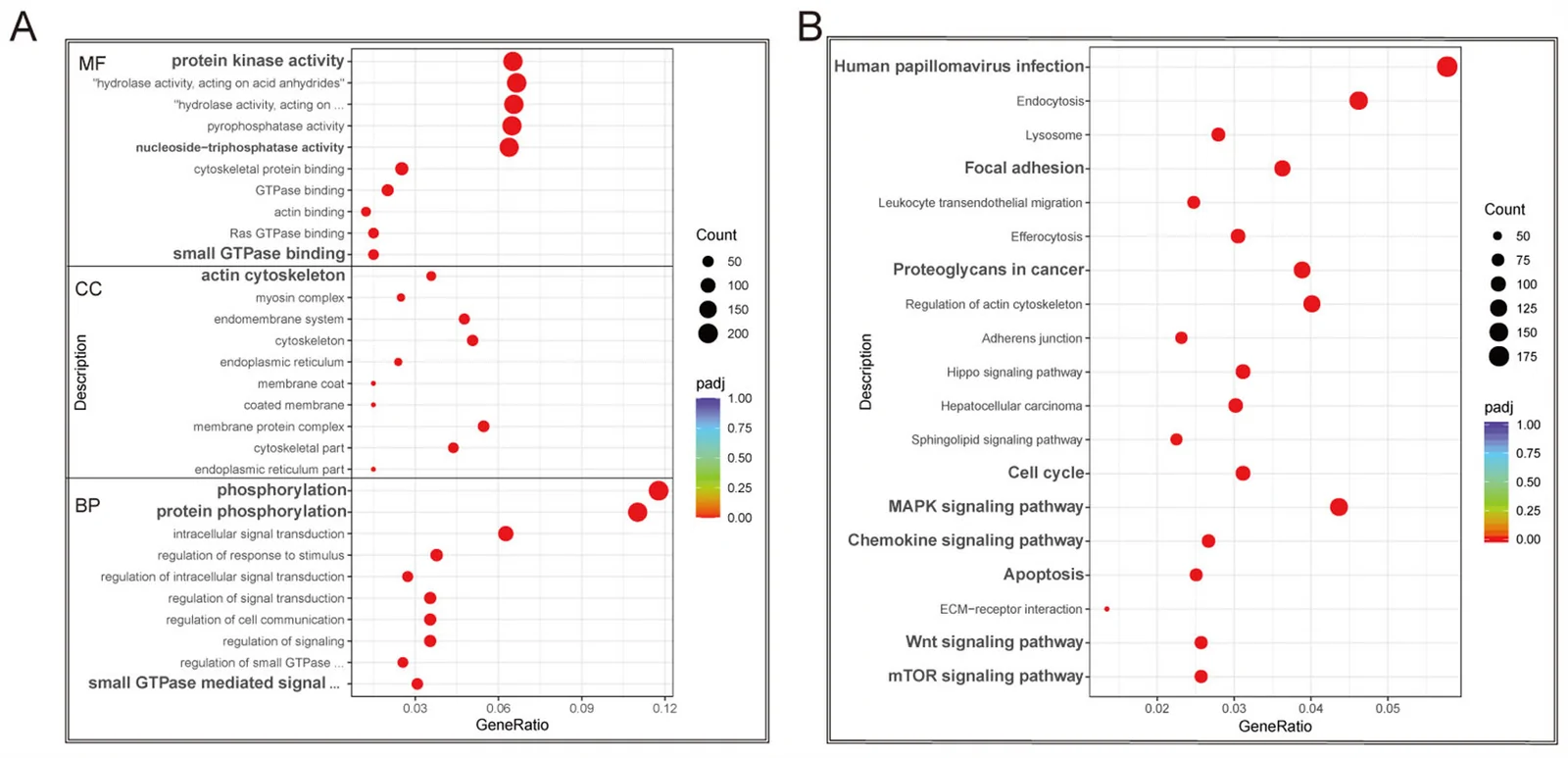
The GO and KEGG enrichment analysis of DEGs in transcriptome sequencing analysis. **(A)** GO enrichment analysis of DEGs in molecular function, cellular component, and biological process. **(B)** KEGG pathway enrichment analysis was performed using the Novogene cloud platform based on our RNA-seq data. The KEGG database is developed by Kanehisa Laboratories (29, 30), and permission for the use of KEGG pathway maps has been obtained from Kanehisa Laboratories.

KEGG pathway analysis further further highlighted potential oncogenic reprogramming, with human papillomavirus (HPV) infection emerging as the top enriched pathway, indicating widespread dysregulation of cell cycle, apoptosis, and immune-related pathways. This was accompanied by enrichment of pathways governing core cancer hallmarks, including cell cycle, evasion of apoptosis, and key signaling cascades such as MAPK and Wnt-related pathways, as inferred from the Proteoglycans in cancer and Focal adhesion pathways. Pathways involved in tumor microenvironment remodeling were also significantly enriched. These included pathways related to cell-matrix interaction, such as Focal adhesion and ECM-receptor interaction; immune modulation, including the Chemokine signaling pathway; and metabolic reprogramming, such as the mTOR signaling pathway (Figure 3B).

Collectively, these transcriptomic results depict a tumor characterized by dysregulated host pathways associated with viral infection, dysregulated kinase signaling, and critically, small GTPase-mediated cytoskeletal reorganization, apoptosis dysregulation, and extensive microenvironmental crosstalk (e.g., focal adhesion, ECM-receptor interaction). These molecular trends appear consistent with the histopathological findings of Pan-CK positivity and Vimentin expression in the tumor tissue, strongly suggesting an epithelial neoplasm undergoing EMT, thereby offering a comprehensive functional framework for understanding the molecular drivers of tumorigenesis in this case.

## WGS analysis reveals the genomic variant landscape between tumor and adjacent tissues

To explore the individual the genomic alterations underlying tumor development, we performed WGS on matched tumor and adjacent non-tumor tissues from the same individual. Sequencing achieved depths of 54.9 × (tumor) and 26.4 × (control tissue), corresponding to about 1.16 billion and 541 million reads, respectively (Table 1). Initial variant calling against the red deer reference genome identified approximately 1.05 million SNPs in each sample (Table 1; Figure 4A). It is crucial to note that these variants primarily represent germline differences, which are inherent genetic distinctions between the genome of this Milu deer individual and the reference genome, and are therefore shared between the tumor and its matched normal tissue. Their genomic distribution was similar in both samples, predominantly located in intergenic (~55%) and intronic (~41%) regions (Figure 4B). Within these exonic regions, the tumor tissue exhibited a slightly higher proportion of nonsynonymous variants at 35.04% and stop-gain variants at 0.29%, compared to 34.78% and 0.28% in the control tissue, respectively, alongside a corresponding reduction in synonymous variants from 62.61% in the control to 62.28% in the tumor (Figure 4B). This suggests a modest enrichment of protein-altering and truncating mutations in the tumor genome, potentially contributing to tumorigenesis.

**Table 1:** Data from DNBSEQ-T7 and sequence assembly.

Sample	# of Reads	# of Reads Aligned	# of Reads Not Aligned	Coverage	Total # of SNP
Adjacent non-tumor tissue	541,098,486	537,304,096	3,794,390	26.41 ×	1,048,362
Tumor	1,161,005,594	1,153,925,978	7,079,616	54.95 ×	1,048,571

**Figure 4:**
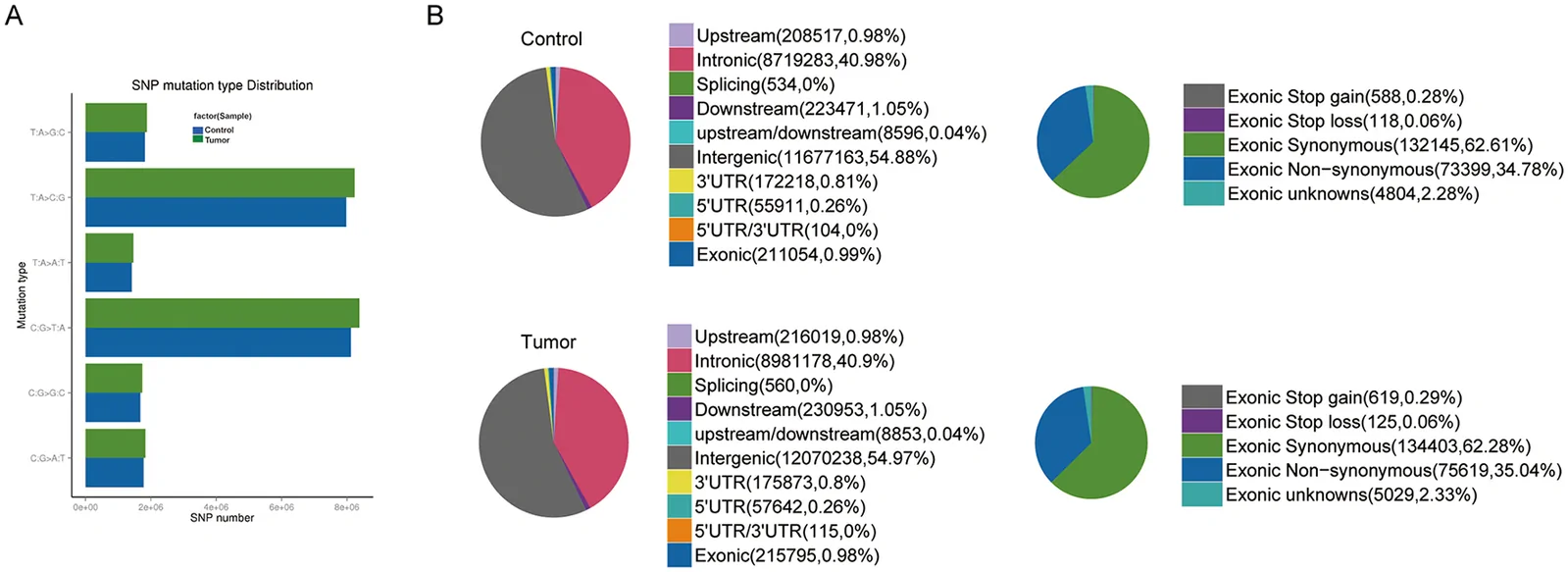
Genomic landscape and functional enrichment analysis from WGS. **(A)** The number of different variant types identified in the tumor and control tissues. **(B)** Stacked bar plots displaying the genomic distribution of SNPs in the tumor and control tissues.

To pinpoint somatic mutations specific to the tumorigenesis process, we performed a comparative analysis between the paired samples. We focused on variants present in the tumor tissue but absent in the matched normal tissue. After applying stringent filters (Alt_depth_tumor ≥ 10, VAF_tumor > 0.10, coding sequence location, non-synonymous impact), we isolated a high-confidence set of 114 tumor-specific somatic mutations (Table 2). Collectively, these analyses reveal both the germline SNP background and tumor-specific somatic mutations in this Milu deer individual, providing a foundation for identifying potential driver alterations.

**Table 2:** High-confidence tumor-specific somatic mutations identified by WGS.

Chromosome	Position	Ref	Alt	Type of mutation	Tumor_ Depth (Ref,Alt)	Tumor_ DP	Normal_ Depth (Ref,Alt)	Normal_ DP	Tumor_ VAF	Normal_ VAF	Tumor_ GT	Normal_ GT	gene_ name
NC_057386.1	10650896	A	C	nonsynonymous SNV	55|17	72	14|3	17	0.236	0.176	0/1	0/0	RNF213
NC_057386.1	10650901	G	A	nonsynonymous SNV	55|16	71	12|3	15	0.225	0.2	0/1	0/0	RNF213
NC_057386.1	21096010	T	A	nonsynonymous SNV	38|11	49	15|1	16	0.224	0.062	0/1	0/0	LOC122442006
NC_057386.1	21096672	G	A	nonsynonymous SNV	26|10	36	12|1	13	0.278	0.077	0/1	0/0	LOC122442006
NC_057386.1	31122616	T	C	nonsynonymous SNV	14|12	30	12|3	15	0.462	0.2	0/1	0/0	LOC122445049, LOC122445150
NC_057386.1	33545006	G	A	nonsynonymous SNV	37|13	50	17|1	18	0.26	0.056	0/1	0/0	PHF23
NC_057386.1	33545018	A	G	nonsynonymous SNV	38|11	49	17|1	18	0.224	0.056	0/1	0/0	PHF23
NC_057386.1	36366493	G	A	nonsynonymous SNV	39|18	57	19|6	25	0.316	0.24	0/1	0/0	LOC122447462
NC_057386.1	36410748	G	C	nonsynonymous SNV	31|10	41	20|4	24	0.244	0.167	0/1	0/0	LOC122447346
NC_057386.1	65632690	C	T	nonsynonymous SNV	50|20	70	31|8	39	0.286	0.205	0/1	0/0	LOC122451708
NC_057387.1	6516687	C	T	nonsynonymous SNV	19|17	36	11|3	14	0.472	0.214	0/1	0/0	C2H1orf226
NC_057387.1	11018189	T	A	nonsynonymous SNV	58|13	71	21|2	23	0.183	0.087	0/1	0/0	LOC122431282
NC_057387.1	11601644	A	C	nonsynonymous SNV	39|34	73	35|6	41	0.466	0.146	0/1	0/0	CD5L
NC_057387.1	11601655	A	G	nonsynonymous SNV	42|47	89	33|9	42	0.528	0.214	0/1	0/0	CD5L
NC_057387.1	11601656	C	T	nonsynonymous SNV	43|47	90	33|9	42	0.522	0.214	0/1	0/0	CD5L
NC_057387.1	14169946	A	T	nonsynonymous SNV	44|12	56	25|2	27	0.214	0.074	0/1	0/0	GON4L
NC_057387.1	20090053	C	T	nonsynonymous SNV	38|14	52	26|3	29	0.269	0.103	0/1	0/0	LOC122434933
NC_057387.1	22553099	G	A	nonsynonymous SNV	40|16	56	13|3	16	0.286	0.188	0/1	0/0	LOC122428549
NC_057387.1	47980876	T	G	nonsynonymous SNV	52|24	76	34|4	38	0.316	0.105	0/1	0/0	LOC122436681, CCDC18
NC_057387.1	113897004	G	T	nonsynonymous SNV	16|17	33	11|2	13	0.515	0.154	0/1	0/0	DUSP28
NC_057388.1	30851740	G	A	nonsynonymous SNV	35|12	47	16|3	19	0.255	0.158	0/1	0/0	ABCB1
NC_057388.1	30924705	C	T	nonsynonymous SNV	65|14	79	42|6	48	0.177	0.125	0/1	0/0	LOC122438231
NC_057388.1	59925882	G	A	nonsynonymous SNV	32|14	46	23|4	27	0.304	0.148	0/1	0/0	LOC122438403,BBS9
NC_057388.1	100029861	T	A	nonsynonymous SNV	32|11	43	18|5	23	0.256	0.217	0/1	0/0	LOC122438750
NC_057388.1	101700684	A	G	nonsynonymous SNV	48|25	73	28|8	36	0.342	0.222	0/1	0/0	LOC122437993
NC_057388.1	112763782	T	C	nonsynonymous SNV	39|27	66	25|5	30	0.409	0.167	0/1	0/0	LOC122438862
NC_057388.1	112763797	C	T	nonsynonymous SNV	42|27	69	25|5	30	0.391	0.167	0/1	0/0	LOC122438862
NC_057388.1	112854186	T	G	nonsynonymous SNV	25|22	47	13|2	15	0.468	0.133	0/1	0/0	LOC122437921
NC_057388.1	112854208	A	C	nonsynonymous SNV	24|26	50	13|2	15	0.52	0.133	0/1	0/0	LOC122437921
NC_057388.1	112854212	A	G	nonsynonymous SNV	25|26	51	13|3	16	0.51	0.188	0/1	0/0	LOC122437921
NC_057389.1	676878	G	C	nonsynonymous SNV	71|17	88	24|3	27	0.193	0.111	0/1	0/0	LOC122439404
NC_057389.1	6953588	G	A	nonsynonymous SNV	36|11	47	12|3	15	0.234	0.2	0/1	0/0	LOC122439480
NC_057389.1	56416095	G	A	nonsynonymous SNV	25|12	37	17|6	23	0.324	0.261	0/1	0/0	LOC122439793
NC_057389.1	56870154	T	C	nonsynonymous SNV	29|10	39	17|2	19	0.256	0.105	0/1	0/0	LOC122439814
NC_057389.1	66077434	A	G	nonsynonymous SNV	48|23	71	26|6	32	0.324	0.188	0/1	0/0	LOC122439238
NC_057389.1	87493488	A	G	nonsynonymous SNV	52|11	63	17|1	18	0.175	0.056	0/1	0/0	CHAF1A
NC_057389.1	93521647	C	T	nonsynonymous SNV	18|13	31	14|5	19	0.419	0.263	0/1	0/0	LOC122440476
NC_057389.1	93625078	G	A	nonsynonymous SNV	52|15	67	19|3	22	0.224	0.136	0/1	0/0	LOC122440468
NC_057389.1	93746406	C	T	nonsynonymous SNV	24|25	49	15|2	17	0.51	0.118	0/1	0/0	LOC122440464
NC_057389.1	93746791	C	A	nonsynonymous SNV	22|19	41	15|2	17	0.463	0.118	0/1	0/0	LOC122440464
NC_057389.1	93757973	T	C	nonsynonymous SNV	17|22	39	21|6	27	0.564	0.222	0/1	0/0	LOC122440464
NC_057389.1	93758069	G	A	nonsynonymous SNV	22|25	47	12|3	15	0.532	0.2	0/1	0/0	LOC122440464
NC_057389.1	93759976	T	A	nonsynonymous SNV	29|24	53	17|5	22	0.453	0.227	0/1	0/0	93759976
NC_057389.1	93759988	G	A	nonsynonymous SNV	28|26	54	19|5	24	0.481	0.208	0/1	0/0	LOC122440464
NC_057389.1	99548915	T	C	nonsynonymous SNV	52|29	81	36|12	48	0.358	0.25	0/1	0/0	ADGRE2
NC_057389.1	100197825	G	T	nonsynonymous SNV	81|23	104	46|9	55	0.221	0.164	0/1	0/0	LOC122439342
NC_057389.1	100197840	G	A	nonsynonymous SNV	87|21	108	51|8	59	0.194	0.136	0/1	0/0	LOC122439342
NC_057389.1	101769395	C	T	nonsynonymous SNV	26|20	46	19|5	24	0.435	0.208	0/1	0/0	LOC122441017
NC_057390.1	88963724	C	T	nonsynonymous SNV	49|14	63	17|6	23	0.222	0.261	0/1	0/0	LOC122442511, LOC122442128
NC_057390.1	89140192	T	A	nonsynonymous SNV	36|22	58	20|4	24	0.379	0.167	0/1	0/0	LOC122442514
NC_057390.1	89140215	T	C	nonsynonymous SNV	34|20	54	23|6	29	0.37	0.207	0/1	0/0	LOC122442514
NC_057391.1	21375393	G	C	nonsynonymous SNV	26|22	48	18|8	26	0.458	0.308	0/1	0/0	LOC122444181
NC_057391.1	21579503	A	G	nonsynonymous SNV	31|27	58	13|2	15	0.466	0.133	0/1	0/0	LOC122444186
NC_057391.1	21599613	C	G	nonsynonymous SNV	62|27	89	24|10	34	0.303	0.294	0/1	0/0	LOC122443241
NC_057391.1	21691090	C	T	nonsynonymous SNV	21|13	34	10|2	12	0.382	0.167	0/1	0/0	LOC122444190
NC_057391.1	21756978	A	G	nonsynonymous SNV	59|22	81	23|4	27	0.272	0.148	0/1	0/0	LOC122444193
NC_057391.1	21804104	A	T	nonsynonymous SNV	89|40	129	53|10	63	0.31	0.159	0/1	0/0	LOC122444197
NC_057391.1	21804106	G	A	nonsynonymous SNV	89|40	129	53|10	63	0.31	0.159	0/1	0/0	LOC122444197
NC_057391.1	21850128	A	G	nonsynonymous SNV	22|11	33	10|4	14	0.333	0.286	0/1	0/0	LOC122443257
NC_057391.1	22349825	G	C	nonsynonymous SNV	63|28	91	35|5	40	0.308	0.125	0/1	0/0	LOC122444212
NC_057391.1	22582944	C	T	nonsynonymous SNV	86|38	124	45|7	52	0.306	0.135	0/1	0/0	LOC122444226
NC_057391.1	22630349	A	G	nonsynonymous SNV	26|14	40	22|1	23	0.35	0.043	0/1	0/0	LOC122444227
NC_057391.1	24365137	G	A	nonsynonymous SNV	32|16	48	26|3	29	0.333	0.103	0/1	0/0	LOC122444256
NC_057391.1	24947470	G	A	nonsynonymous SNV	14|5	19	14|5	19	0.343	0.263	0/1	0/0	LOC122444084
NC_057391.1	25095774	G	A	nonsynonymous SNV	55|16	71	28|2	30	0.225	0.067	0/1	0/0	LOC122442918
NC_057391.1	25190260	G	A	nonsynonymous SNV	10|12	22	13|2	15	0.545	0.133	0/1	0/0	LOC122444271
NC_057391.1	38919194	G	A	nonsynonymous SNV	17|12	29	10|2	12	0.414	0.167	0/1	0/0	STARD9
NC_057391.1	38919932	A	G	nonsynonymous SNV	24|21	45	25|5	30	0.467	0.167	0/1	0/0	STARD9
NC_057391.1	38919933	T	C	nonsynonymous SNV	24|21	45	25|5	30	0.467	0.167	0/1	0/0	STARD9
NC_057391.1	62954403	G	C	nonsynonymous SNV	43|15	58	20|3	23	0.259	0.13	0/1	0/0	LGALS3
NC_057392.1	21935364	G	T	nonsynonymous SNV	17|12	29	15|2	17	0.414	0.118	0/1	0/0	TSPEAR, LOC122445416
NC_057392.1	34648335	A	G	nonsynonymous SNV	106|27	133	57|10	67	0.203	0.149	0/1	0/0	NEPRO, LOC122445545
NC_057392.1	34648719	A	C	nonsynonymous SNV	88|39	127	50|12	62	0.307	0.194	0/1	0/0	NEPRO, LOC122445545
NC_057393.1	9506283	G	A	nonsynonymous SNV	33|10	43	16|0	16	0.233	0	0/1	0/0	DMBT1
NC_057393.1	54639075	C	G	nonsynonymous SNV	52|11	63	24|5	29	0.175	0.172	0/1	0/0	ZNF239
NC_057393.1	55802603	T	C	nonsynonymous SNV	53|17	70	20|7	27	0.243	0.259	0/1	0/0	LOC122446294
NC_057393.1	68313327	C	T	nonsynonymous SNV	45|16	61	25|2	27	0.262	0.074	0/1	0/0	DDX21
NC_057393.1	79811105	T	G	nonsynonymous SNV	48|11	59	20|3	23	0.186	0.13	0/1	0/0	COG2
NC_057393.1	82795078	G	C	nonsynonymous SNV	25|10	35	15|1	16	0.286	0.062	0/1	0/0	ZP4
NC_057393.1	86988285	T	C	nonsynonymous SNV	29|12	41	23|4	27	0.293	0.148	0/1	0/0	MAP3K21
NC_057394.1	14946843	C	T	nonsynonymous SNV	27|15	42	11|3	14	0.357	0.214	0/1	0/0	LOC122447477, LOC122447478
NC_057395.1	9480135	T	C	nonsynonymous SNV	41|14	55	36|3	39	0.255	0.077	0/1	0/0	LOC122447855, LOC122447854
NC_057395.1	10198621	T	A	nonsynonymous SNV	31|34	65	23|8	31	0.523	0.258	0/1	0/0	LOC122447855
NC_057395.1	10222360	A	G	nonsynonymous SNV	44|15	59	21|3	24	0.254	0.125	0/1	0/0	LOC122447858
NC_057395.1	52221629	G	T	nonsynonymous SNV	42|12	54	21|5	26	0.222	0.192	0/1	0/0	SIRPA
NC_057395.1	55503900	C	G	nonsynonymous SNV	40|18	58	22|3	25	0.31	0.12	0/1	0/0	LOC122448632
NC_057395.1	72003276	G	T	nonsynonymous SNV	35|18	53	12|2	14	0.34	0.143	0/1	0/0	LOC122448931
NC_057395.1	75965906	T	C	nonsynonymous SNV	23|21	44	13|4	17	0.477	0.235	0/1	0/0	LOC122448179
NC_057395.1	81621161	T	C	nonsynonymous SNV	26|10	36	24|2	26	0.278	0.077	0/1	0/0	LOC122448950
NC_057396.1	5882693	G	A	nonsynonymous SNV	18|10	28	10|5	15	0.357	0.333	0/1	0/0	TRPC6, LOC122450043
NC_057396.1	38065886	G	A	nonsynonymous SNV	27|10	37	13|2	15	0.27	0.133	0/1	0/0	TRPC6, LOC122450043
NC_057396.1	42481405	A	C	nonsynonymous SNV	46|20	66	20|4	24	0.303	0.167	0/1	0/0	LOC122449846, LOC122449250
NC_057396.1	42519130	G	A	nonsynonymous SNV	60|14	74	34|1	35	0.189	0.029	0/1	0/0	LOC122449250
NC_057396.1	42597122	C	T	nonsynonymous SNV	57|23	80	32|6	38	0.287	0.158	0/1	0/0	LOC122449829
NC_057396.1	44128304	A	G	nonsynonymous SNV	31|10	41	20|1	21	0.244	0.048	0/1	0/0	LOC122449883
NC_057396.1	44270817	G	A	nonsynonymous SNV	69|25	94	26|3	29	0.266	0.103	0/1	0/0	LOC122450100
NC_057396.1	44271382	C	T	nonsynonymous SNV	43|18	61	31|3	34	0.295	0.088	0/1	0/0	LOC122450100
NC_057396.1	45721894	T	C	nonsynonymous SNV	34|17	51	16|0	16	0.333	0	0/1	0/0	LOC122450288
NC_057396.1	46217276	A	T	nonsynonymous SNV	49|27	76	30|5	35	0.355	0.143	0/1	0/0	LOC122450606
NC_057396.1	49499553	C	T	nonsynonymous SNV	60|17	77	21|3	34	0.221	0.125	0/1	0/0	LOC122450606
NC_057396.1	50125849	C	T	nonsynonymous SNV	83|20	103	28|4	32	0.194	0.125	0/1	0/0	LOC122449554
NC_057396.1	51294110	G	A	nonsynonymous SNV	27|12	39	21|5	26	0.308	0.192	0/1	0/0	TSKU
NC_057396.1	71950249	C	T	nonsynonymous SNV	10|13	23	10|5	15	0.565	0.333	0/1	0/0	LOC122449874
NC_057396.1	72383888	C	G	nonsynonymous SNV	27|16	43	14|4	18	0.372	0.222	0/1	0/0	LOC122450281
NC_057396.1	72439377	G	C	nonsynonymous SNV	40|15	55	19|3	22	0.273	0.136	0/1	0/0	LOC122450545
NC_057396.1	72577538	A	C	nonsynonymous SNV	26|11	37	16|2	18	0.297	0.111	0/1	0/0	LOC122450505
NC_057396.1	72648603	A	C	nonsynonymous SNV	74|23	97	29|3	32	0.237	0.094	0/1	0/0	LOC122450544
NC_057396.1	72838803	G	C	nonsynonymous SNV	36|18	54	20|5	25	0.333	0.2	0/1	0/0	LOC122450515
NC_057396.1	72977221	T	G	nonsynonymous SNV	25|21	46	20|7	27	0.457	0.259	0/1	0/0	LOC122450529, LOC122450481
NC_057396.1	72977223	G	C	nonsynonymous SNV	25|21	46	20|6	26	0.457	0.231	0/1	0/0	LOC122450529, LOC122450481
NC_057396.1	74182719	C	G	nonsynonymous SNV	27|11	38	13|2	15	0.289	0.133	0/1	0/0	LOC122449360
NC_057396.1	74216964	A	C	nonsynonymous SNV	30|23	53	20|3	23	0.434	0.13	0/1	0/0	LOC122450493
NC_057396.1	74227213	T	A	nonsynonymous SNV	100|37	137	63|12	75	0.27	0.16	0/1	0/0	LOC122450492
NC_057397.1	10045642	G	C	nonsynonymous SNV	28|10	38	12|2	14	0.263	0.143	0/1	0/0	PLEC

## Joint analysis of WGS and RNA-Seq identifies putative tumor-associated genes in tumorigenesis

To prioritize candidate genes with the strongest support for a driver role, we integrated the genomic and transcriptomic datasets. We intersected the list of 114 genes harboring high-confidence, tumor-specific nonsynonymous somatic mutations (from WGS) with the list of 6,537 DEGs (from RNA-seq). This integration identified ten genes that were both somatically mutated and significantly differentially expressed: namely, *GON4L, DUSP28, BBS9, CHAF1A, LGALS3, DMBT1, DDX21, COG2, SIRPA* and *TSKU* (Table 3). These are thus highlighted as prime candidates that may contribute to tumor development.

**Table 3:** Ten putative tumor-associated genes identified by integrated WGS and RNA-seq.

Gene name	Location	gene ID	M (log2 fold change)	Ref	Alt
*GON4L*	Nucleus (red deer)	122677286	1.71061810484873	A	T
*DUSP28*	-	122676474	−2.45208585706093	G	T
*BBS9*	Membrane (red deer)	122674871	2.81875451436382	G	A
*CHAF1A*	Nucleus (red deer)	122699674	3.41718765488243	A	G
*LGALS3*	Cytoplasm;Nucleus; Sereted; (White-tailed deer)	122699674	10.3141220948697	G	C
*DMBT1*	Membrane (White-tailed deer)	122708800	14.5132610443726	G	A
*DDX21*	Cytoplasm; Nucleus (White-tailed deer)	122709029	2.59636209302738	C	T
*COG2*	-	122708967	2.19893529087014	T	G
*SIRPA*	Membrane (Human)	122681212	2.76563532348915	G	T
*TSKU*	extracellular matrix (White-tailed deer)	122697324	5.66032951530482	G	A

The direction of expression change for these genes revealed distinct functional patterns. Notably, *DMBT1* (M = +14.51) and *LGALS3* (M = +10.31) were among the most strongly upregulated genes, consistent with roles in tumor metastasis and immunosuppression, respectively. In addition, *TSKU* (M = +5.66), *CHAF1A* (M = +3.42), and *BBS9* (M = +2.82) also exhibited apparent upregulation trends, which may theoretically relate to extracellular matrix remodeling, chromatin assembly, and ciliary signaling. In contrast, *DUSP28* (M = −2.45) showed the most pronounced downregulation trend, potentially indicating loss of its regulatory function in MAPK signaling. This integrated mutational and transcriptional profile provides a nuanced view of the oncogenic landscape in Milu deer.

## Discussion

This exploratory case study represents the first integrated genomic, transcriptomic, and histopathological characterization of a single facial tumor in the Milu deer, aiming to enrich the baseline pathological data for this species. Histopathological evaluation defined this neoplasm as an epithelial tumor (Pan-CK positive) with substantial mesenchymal components (Vimentin positive), definitively ruling out a neural crest origin (S100/SOX10 negative). Building upon this pathological framework, our matched-tissue multi-omics profiling yielded a high-confidence set of 114 somatic mutations and 6,537 DEGs. Joint analysis of WGS and RNA-Seq identified ten putative tumor-associated genes, including *LGALS3, DMBT1*, and *CHAF1A*, alongside a characterized “immune-cold” histopathological profile, providing an unprecedented molecular baseline for understanding tumorigenesis in this species.

The histopathological evidence of an epithelial origin coupled with mesenchymal features is strongly corroborated by our transcriptomic data. We observed significant enrichment of pathways directly associated with cytoskeletal reorganization, small GTPase binding, focal adhesion, and ECM-receptor interaction (Figures 3A, B). These molecular pathways are widely recognized hallmarks of EMT, a crucial process in tumor progression where epithelial cells lose polarity, acquire mesenchymal characteristics, and enhance their migratory and invasive capabilities (31). Consequently, the dual immunophenotype (Pan-CK+/Vimentin+) combined with this distinct transcriptomic signature indicates a highly plastic and aggressive tumor microenvironment. The observed lack of CD3+ and CD68+ immune cell infiltration (Figures 1F–G) suggests a state of immune exclusion. This “immune-cold” phenotype may be intrinsically linked to the active EMT process identified in our transcriptomic data; the remodeling of the extracellular matrix (ECM) and the acquisition of mesenchymal features are known to create physical and chemical barriers that impede the recruitment and infiltration of host immune cells (32). This interaction between mesenchymal-like tumor cells and the absence of a cellular immune response likely facilitates the tumor's ability to bypass local surveillance.

Functional enrichment analysis further delineated a complex network of dysregulated pathways driving this malignant transition. Notably, the prominent enrichment of the “HPV infection” pathway aligns seamlessly with the tumor's epithelial classification. HPV primarily infects epithelial cells and is a well-established driver of various epithelial cancers in humans and other species (33). Rather than indicating an active infection, the enrichment of this pathway mainly reflects the disruption of host cellular processes such as cell cycle progression, evasion of apoptosis, and immune modulation, which are processes commonly manipulated during viral-driven cancer development. This is further supported by the concurrent activation of PI3K-Akt, MAPK, and mTOR cascades, which together orchestrate a state of enhanced proliferation and metabolic reprogramming. The convergence of these signals, alongside actin cytoskeleton remodeling, eflects a profound collapse of host cellular homeostasis. We interpret the enrichment of the “HPV infection” pathway not as evidence of viral involvement, but as a biological artifact resulting from the severe dysregulation of core pathways governing the cell cycle and genomic stability, which are evolutionarily conserved and typically exploited during viral oncogenesis. This finding is particularly intriguing when contextualized with what is known about wild cervids, where papillomaviruses such as CcaPV1 and CePV1 are classical drivers of cutaneous fibropapillomas and facial masses (10, 11) Although our transcriptomic and genomic data suggest that the Milu deer tumor represents an endogenous molecular “phenocopy” (where host network collapse mimics the functional landscape of viral carcinogenesis without active viral replication), the widespread prevalence of oncogenic papillomaviruses across European and North American deer populations underscores the urgent need for broader, systematic viral surveillance in this endangered population to definitively distinguish sporadic non-communicable neoplasms from potential viral outbreaks.

At the gene level, our comparative analysis with established human oncology literature provides mechanistic insights into the observed molecular alterations. Several identified candidate genes are established drivers of proliferation and metastasis. For instance, DMBT1 showed the most pronounced upregulation, suggesting a potential pro-metastatic role consistent with its function in promoting breast cancer liver metastasis (34–36). Similarly, the marked upregulation of LGALS3, which is known to foster immunosuppressive tumor microenvironments and inhibit T-cell infiltration, provides a plausible molecular driver for the lack of CD3+ T cells observed in our IHC analysis. Similarly, the upregulation of *SIRPA*, a key “don't eat me” signaling molecule, aligns with the absence of CD68+ macrophages within the tumor mass (37–42), correlates directly with the macroscopic observation of the tumor's expansile and infiltrative growth pattern. Additionally, the upregulation of TSKU and CHAF1A further supports a highly invasive and proliferative phenotype, given their respective associations with EMT (43, 44) and chromatin assembly (45, 46). Other candidates, including GON4L, SIRPA, and DUSP28, also exhibited expression changes consistent with their established roles in tumor growth (47), immune modulation (48, 49) and MAPK signaling (50–55). Collectively, the identification of these oncogenic and tumor-suppressive counterparts in the Milu deer establishes a robust molecular foundation for future targeted investigations. The significance of these findings extends beyond this individual pathology to comparative oncology and wildlife conservation. The identification of an “immune-cold” tumor in the Milu deer is particularly significant given the species' comparatively low incidence of carcinogenesis [~5 times lower than other mammals (26)]. This suggests that while Milu deer may possess robust systemic tumor-suppressive mechanisms, specific malignancies can still arise by establishing a potent immunosuppressive niche that effectively shields the tumor from the host's baseline immune defenses. Identifying specific molecular targets, such as *SIRPA* and *LGALS3*, establishes a preliminary foundation for potential checkpoint inhibition or targeted therapies in high-value captive animals. Moreover, the observed metabolic reprogramming and dysregulation of host pathways, annotated in KEGG as viral infection pathways (e.g., HPV infection), reflect profound dysregulation of highly conserved host cell cycle and apoptotic networks, such as p53 and Rb signaling, which confirms the absence of an actual viral etiology, as Milu deer are not known hosts for HPV. Instead, these pathway enrichments should be viewed as a molecular “phenocopy” where endogenous dysregulation of the p53 and Rb networks mimics the functional landscape of viral-driven cancers. Given that European and North American cervids frequently present with clinically similar lesions driven by endemic papillomaviruses (10, 11), establishing this molecular baseline is paramount. This interpretation provides a critical health-monitoring baseline for bottlenecked populations, where reduced genetic diversity may amplify susceptibility to environmental stressors. Most intriguingly, the susceptibility of Milu deer somatic tissue to tumorigenesis contrasts starkly with the species remarkable resistance to cancer during rapid antler regeneration. We hypothesize that the oncogenic networks identified here are effectively suppressed within the antler microenvironment, presenting a unique biological paradox.

Despite these novel insights, our findings must be interpreted within the context of several limitations. Primarily, as this study is based on a single sporadic case (*N* = 1), the results must be viewed exclusively as an exploratory, hypothesis-generating case report rather than definitive evidence of species-wide disease mechanisms. Second, regarding the transcriptomic profiling, the lack of biological replicates (at least *n* = 3) means that conventional statistical identification of DEGs is not valid. As such, the transcriptomic findings must be framed strictly as individual observational trends rather than definitive statistical biomarkers, and biological noise cannot be statistically eliminated. Third, the absence of a highly annotated, species-specific Milu deer reference genome necessitates mapping to closely related species, which inherently introduces interpretation bias in genomic and transcriptomic analyses. While the use of a *C. elaphus* reference genome introduces potential mapping biases, the risk of false-positive somatic calls arising from fixed species-level divergence was mitigated by our matched-pair analysis, which subtracts shared genomic backgrounds. Therefore, these multi-omics profiles serve exclusively as preliminary baseline data to inform future wildlife disease surveillance rather than generalizable disease markers.

In conclusion, this study presents the first integrated molecular and pathological characterization of a single Milu deer facial tumor, revealing a mechanism driven by dysregulated cell cycles, metabolic reprogramming, and kinase signaling, alongside a core set of ten putative tumor-associated genes. Future research should prioritize the assembly of a *de novo* Milu deer genome to enhance variant calling precision. Furthermore, because the DEG analysis was inherently limited by the absence of biological replicates, the transcriptomic profile may reflect individual idiosyncrasies. Therefore, independent *in vitro* and *in vivo* functional validation of these candidate genes across multiple samples is required to confirm their roles in driving malignant phenotypes. Ultimately, contrasting these tumor-prone somatic profiles with the naturally tumor-resistant tissues of regenerating antlers represents a compelling frontier for uncovering evolutionarily conserved tumor-suppressive mechanisms.

## Data Availability

The datasets presented in this study can be found in online repositories. The names of the repository/repositories and accession number(s) can be found at: https://ngdc.cncb.ac.cn/gsa/, CRA032811(RNA-seq) and CRA033238(WGS).

## Supplementary material

Supplementary Table S1 Primer sequences used in this study.

Supplementary Table S2 Summary of RNA-seq quality control metrics.

Supplementary Table S3 Comprehensive list of DEGs between tumor and adjacent non-tumor tissues. Mean expression in tumor and adjacent tissues (Tumor_mean, Control_mean), M value (log2 fold change; positive indicates upregulation, negative indicates downregulation), D value (absolute expression difference, typically on a log scale), prob value (probability of differential expression; values closer to 1 indicate higher confidence), and ranking value (composite metric integrating multiple statistics).

Supplementary Figure S1 Representative macroscopic photograph of the resected facial tumor and adjacent non-tumor tissue.

## References

[1] Zhang Y Li Z Song X Xiao G He L Bai J . Genetic diversity of trichomonads from Milu deer (*Elaphurus davidianus*) in China. Parasit. (2025) 32:23. doi: 10.1051/parasite/2025015 40214164 PMC11987501

[2] Agrimi U Morelli L Di Guardo G. Squamous cell carcinoma of the skin in a Père David's Deer (*Elaphurus davidianus*). J Wildl Dis. (1993) 29:616–7. doi: 10.7589/0090-3558-29.4.616 8258867

[3] Cheng Z Zhang Y Liu W Zhong Z Bai J Cheng K . Population dynamics and the role of protected areas in China's Milu deer (*Elaphurus davidianus*) rewilding. Sci Rep. (2025) 15:188. doi: 10.1038/s41598-024-84456-3 39747309 PMC11697174

[4] Cheng Z Zhang H Ma J Feng C Liu W Zhong Z . Behavioral shifts of reintroduced Milu deer (*Elaphurus davidianus*) in East Dongting lake of China. Sci Rep. (2025) 15:34833. doi: 10.1038/s41598-025-21345-3 41057647 PMC12504694

[5] Chen Y Yan Y Gao Y Li Y Zhang K Zhou M . An outbreak of tuberculosis in endangered northern pig-tailed Macaques (*Macaca leonina*) and Milu deer (*Elaphurus davidianus*) from a zoo in China. Vet Med Sci. (2023) 9:992–8. doi: 10.1002/vms3.1014 36626281 PMC10029874

[6] Woods GM Lyons AB Bettiol SS. A devil of a transmissible cancer. Trop Med Infect Dis. (2020) 5:50. doi: 10.3390/tropicalmed5020050 32244613 PMC7345153

[7] Espejo C Patchett AL Wilson R Lyons AB Woods GM. Challenges of an emerging disease: the evolving approach to diagnosing devil facial tumor disease. Pathogens. (2021) 11:27. doi: 10.3390/pathogens11010027 35055975 PMC8780694

[8] Xiong Z Ling S Li C Deng L He M Wang C . Case report: a study on the pathological diagnosis of oral fibrosarcoma in a giant panda. Front Vet Sci. (2025) 11:1500679. doi: 10.3389/fvets.2024.1500679 39968290 PMC11832535

[9] McLaughlin A Tripp C Bertram C Kiupel M Thaiwong T Reavill D. Cutaneous squamous cell carcinomas in domestic rabbits (*Oryctolagus cuniculus*): 39 Cases (1998–2019). J Exot Pet Med. (2021) 39:38–50. doi: 10.1053/j.jepm.2021.06.001

[10] Erdélyi K Dencso L Lehoczki R Heltai M Sonkoly K Csányi S . Endemic papillomavirus infection of roe deer (*Capreolus capreolus*). Vet Microbiol. (2009) 138:20–6. doi: 10.1016/j.vetmic.2009.02.002 19264429

[11] Scagliarini A Gallina L Battilani M Turrini F Savini F Lavazza A . Cervus elaphus papillomavirus (CePV1): new insights on viral evolution in deer. Vet Microbiol. (2013) 165:252–9. doi: 10.1016/j.vetmic.2013.03.012 23578708

[12] Campos Krauer JM Wisely SM. Facts about wildlife diseases: warts in white-tailed deer (*Cutaneous Fibromas*). EDIS. (2024) WEC465. doi: 10.32473/edis-uw523-2024

[13] Zhang Y Bai J Zhu A Chen R Xue D Zhong Z . Reversing extinction in China's Père David's deer. Science. (2021) 372:6539. doi: 10.1126/science.abg6237 33574204

[14] Kausar MA Alshammari KF Alenazi F Anwar S Khalifa AM Ginawi T . RAD51 and PALB2 in precision oncology: clinical implications for HRD associated breast and ovarian cancer. Int J Oncol. (2025) 67:65. doi: 10.3892/ijo.2025.5771 40613200 PMC12270392

[15] Vigneshwaran G Hasan QA Kumar R Eranki A. Analysis of single-nucleotide polymorphisms in genes associated with triple-negative breast cancer. Front Genet. (2022) 6:1071352. 36561320 10.3389/fgene.2022.1071352 PMC9763624

[16] Sachidanandam R Weissman D Schmidt SC Kakol JM Stein LD Marth G . A map of human genome sequence variation containing 1.42 million single nucleotide polymorphisms. Nature. (2001) 409:928–33. doi: 10.1038/35057149 11237013

[17] Sutherland HG Albury CL Griffiths LR. Advances in genetics of migraine. J Headache Pain. (2019) 21:72. doi: 10.1186/s10194-019-1017-9 31226929 PMC6734342

[18] Xu Z Ji C Zhang Y Zhang Z Nie Q Xu J . Combination analysis of genome-wide association and transcriptome sequencing of residual feed intake in quality chickens. BMC Genomics. (2016) 9:594. doi: 10.1186/s12864-016-2861-5 27506765 PMC4979145

[19] Bello SF Adeola AC Nie Q. The study of candidate genes in the improvement of egg production in ducks–a review. Poult Sci. (2022) 101:101850. doi: 10.1016/j.psj.2022.101850 35544958 PMC9108513

[20] Liu C Joehanes R Ma J Xie J Yang J Wang M . Integrating whole genome and transcriptome sequencing to characterize the genetic architecture of isoform variation and its implications for health and disease. medRxiv. (2024) 6:2024.12.04.24318434. doi: 10.1101/2024.12.04.24318434 41274869 PMC12660940

[21] Cai D Wang Z Zhou Z Lin D Ju X Nie Q. Integration of transcriptome sequencing and whole genome resequencing reveal candidate genes in egg production of upright and pendulous-comb chickens. Poult Sci. (2023) 102:102504. doi: 10.1016/j.psj.2023.102504 36739803 PMC9932115

[22] Yang Z Liu Y Luo J Xiao C Yuan H Du W . Integrating whole-genome re-sequencing and transcriptome data to reveal the molecular mechanism of TBX5 gene regulating feathered feet in Guangxi native chickens. Poult Sci. (2025) 104:104871. doi: 10.1016/j.psj.2025.104871 39923455 PMC12011099

[23] Cuppen E Elemento O Rosenquist R Nikic S M IJ Zaleski ID . Implementation of whole-genome and transcriptome sequencing into clinical cancer care. JCO Precis Oncol. (2022) 6:e2200245. doi: 10.1200/PO.22.00245 36480778 PMC10166391

[24] Wong M Mayoh C Lau LMS Khuong-Quang DA Pinese M Kumar A . Whole genome, transcriptome and methylome profiling enhances actionable target discovery in high-risk pediatric cancer. Nat Med. (2020) 26:1742–53. doi: 10.1038/s41591-020-1072-4 33020650

[25] Wang Y Zhang C Wang N Li Z Heller R Liu R . Genetic basis of ruminant headgear and rapid antler regeneration. Science. (2019) 364:eaav6335. doi: 10.1126/science.aav6335 31221830

[26] Lombard LS Witte EJ. Frequency and types of tumors in mammals and birds of the Philadelphia Zoological Garden. Cancer Res. (1959) 19:127–41. 13629476

[27] Sisoudiya SD Tukachinsky H Keller-Evans RB Schrock AB Huang RSP Gjoerup O . Tissue-based genomic profiling of 300,000 tumors highlights the detection of variants with low allele fraction. NPJ Precis Oncol. (2025) 9:190. doi: 10.1038/s41698-025-00991-w 40527927 PMC12174337

[28] Vanni I Casula M Pastorino L Manca A Dalmasso B Andreotti V . Quality assessment of a clinical next-generation sequencing melanoma panel within the Italian Melanoma Intergroup (IMI). Diagn Pathol. (2020) 15:143. doi: 10.1186/s13000-020-01052-5 33317587 PMC7737361

[29] Kanehisa M Furumichi M Tanabe M Sato Y Morishima K KEGG. new perspectives on genomes, pathways, diseases and drugs. Nucleic Acids Res. (2017) 45:D353–61. doi: 10.1093/nar/gkw1092 27899662 PMC5210567

[30] Kanehisa M Furumichi M Sato Y Matsuura Y Ishiguro-Watanabe M KEGG. biological systems database as a model of the real world. Nucleic Acids Res. (2025) 53:D672–7. doi: 10.1093/nar/gkae909 39417505 PMC11701520

[31] Dongre A Weinberg RA. New insights into the mechanisms of epithelial-mesenchymal transition and implications for cancer. Nat Rev Mol Cell Biol. (2019) 20:69–84. doi: 10.1038/s41580-018-0080-4 30459476

[32] Faisal SM Comba A Varela ML Argento AE Brumley E Abel C . The complex interactions between the cellular and non-cellular components of the brain tumor microenvironmental landscape and their therapeutic implications. Front Oncol. (2022) 12:1005069. doi: 10.3389/fonc.2022.1005069 36276147 PMC9583158

[33] Spindler KG Jakobsen AV Eriksen JG Fokdal L Nordsmark M Thorsen LBJ . The clinical utility of circulating human papillomavirus across squamous cell carcinomas. Acta Oncol. (2025) 64:1–12. doi: 10.2340/1651-226X.2025.41288 39748655 PMC11711493

[34] Tian P Wu Q He D Zhao W Luo L Jia Z . Breast cancer induces CD62L(+) Kupffer cells via DMBT1 to promote neutrophil extracellular trap formation and liver metastasis. Cell Discov. (2025) 11:68. doi: 10.1038/s41421-025-00819-8 40796724 PMC12343785

[35] Wu W Kemp BL Proctor ML Gazdar AF Minna JD Hong WK . Expression of DMBT1, a candidate tumor suppressor gene, is frequently lost in lung cancer. Cancer Res. (1999) 59:1846–51. 10213490

[36] Wang L Tuo H Song Z Li W Peng Y. Reg3A (regenerating family member 3 alpha) acts as a tumor suppressor by targeting DMBT1 (deleted in malignant brain tumors 1) in gastric cancer. Bioengineered. (2021) 12:7644–55. doi: 10.1080/21655979.2021.1981800 34605357 PMC8806639

[37] Wang K Fu S Dong L Zhang D Wang M Wu X . Periplocin suppresses the growth of colorectal cancer cells by triggering LGALS3 (galectin 3)-mediated lysophagy. Autophagy. (2023) 19:3132–50. doi: 10.1080/15548627.2023.2239042 37471054 PMC10621285

[38] Zhao S Zhao R Wang C Ma C Gao Z Li B . HDAC7 drives glioblastoma to a mesenchymal-like state via LGALS3-mediated crosstalk between cancer cells and macrophages. Theranostics. (2024) 14:7072–87. doi: 10.7150/thno.100939 39629136 PMC11610139

[39] Wang H Song X Huang Q Xu T Yun D Wang Y . LGALS3 promotes treatment resistance in glioblastoma and is associated with tumor risk and prognosis. Cancer Epidemiol Biomarkers Prev. (2019) 28:760–9. doi: 10.1158/1055-9965.EPI-18-0638 30341098

[40] Fan Y Song S Li Y Dhar SS Jin J Yoshimura K . Galectin-3 cooperates with CD47 to suppress phagocytosis and T-cell immunity in gastric cancer peritoneal metastases. Cancer Res. (2023) 83:3726–38. doi: 10.1158/0008-5472.CAN-23-0783 37738407 PMC10843008

[41] Yang D Sun X Moniruzzaman R Wang H Citu C Zhao Z . Genetic deletion of galectin-3 inhibits pancreatic cancer progression and enhances the efficacy of immunotherapy. Gastroenterology. (2024) 167:298–314. doi: 10.1053/j.gastro.2024.03.007 38467382 PMC11972442

[42] Zheng P Xiao Y Wu Z Wang Q Lv Y Niu W . Tumor-secreted galectin-3 suppresses antitumor response by inducing IL-10^+^ B cells. J Immunother Cancer. (2025) 31:*e*011445. doi: 10.1136/jitc-2024-011445 40449954 PMC12142118

[43] Yamada T Ohta K Motooka Y Fujino K Kudoh S Tenjin Y . Significance of Tsukushi in lung cancer. Lung Cancer. (2019) 131:104–11. doi: 10.1016/j.lungcan.2019.03.024 31027686

[44] Huang H Zhang D Fu J Zhao L Li D Sun H . Tsukushi is a novel prognostic biomarker and correlates with tumor-infiltrating B cells in non-small cell lung cancer. Aging. (2021) 13:4428–51. doi: 10.18632/aging.202403 33428594 PMC7906171

[45] Wu Z Cui F Yu F Peng X Jiang T Chen D . Up-regulation of CHAF1A, a poor prognostic factor, facilitates cell proliferation of colon cancer. Biochem Biophys Res Commun. (2014) 27:208–15. doi: 10.1016/j.bbrc.2014.05.006 24845563

[46] Yan W Shi X Wang H Liao A Yang W. Aberrant SPOP-CHAF1A ubiquitination axis triggers tumor autophagy that endows a therapeutical vulnerability in diffuse large B cell lymphoma. J Transl Med. (2022) 30:296. doi: 10.1186/s12967-022-03476-7 35773729 PMC9248129

[47] Agarwal N Dancik GM Goodspeed A Costello JC Owens C Duex JE . GON4L drives cancer growth through a YY1-androgen receptor-CD24 Axis. Cancer Res. (2016) 76:5175–85. doi: 10.1158/0008-5472.CAN-16-1099 27312530 PMC5010501

[48] Zhou Z Chen MM Luo Y Mojumdar K Peng X Chen H . Tumor-intrinsic SIRPA promotes sensitivity to checkpoint inhibition immunotherapy in melanoma. Cancer Cell. (2022) 40:1324.e8–40.e8. doi: 10.1016/j.ccell.2022.10.012 36332624 PMC9669221

[49] Song A Yang QC Wang WD Wang S Li H Wu L . Tumor-intrinsic SIRPA drives pyroptosis evasion in head and neck cancer. J Dent Res. (2025) 104:645–55. doi: 10.1177/00220345241305590 39904995

[50] Wang D Han S Peng R Jiao C Wang X Han Z . DUSP28 contributes to human hepatocellular carcinoma via regulation of the p38 MAPK signaling. Int J Oncol. (2014) 45:2596–604. doi: 10.3892/ijo.2014.2653 25230705

[51] Lee J Hun Yun J Lee J Choi C Hoon Kim J. Blockade of dual-specificity phosphatase 28 decreases chemo-resistance and migration in human pancreatic cancer cells. Sci Rep. (2015) 27:12296. doi: 10.1038/srep12296 26212664 PMC4515742

[52] Lee J Lee J Yun JH Choi C Cho S Kim SJ . Autocrine DUSP28 signaling mediates pancreatic cancer malignancy via regulation of PDGF-A. Sci Rep. (2017) 7:12760. doi: 10.1038/s41598-017-13023-w 28986588 PMC5630619

[53] Lee J Lee J Kim JH. Scattered DUSP28 is a novel biomarker responsible for aggravating malignancy via the autocrine and paracrine signaling in metastatic pancreatic cancer. Cancer Lett. (2019) 28:216454. doi: 10.1101/313957 30902562

[54] Zhang J Guo Y. DUSP28 promotes cell proliferation, migration, and invasion by Akt/β-catenin/Slug axis in breast cancer. Acta Biochim Pol. (2022) 69:711–7. doi: 10.18388/abp.2020_5843 36520087

[55] Lee J Lee J Yun JH Jeong DG Kim JH. DUSP28 links regulation of Mucin 5B and Mucin 16 to migration and survival of AsPC-1 human pancreatic cancer cells. Tumor Biol. (2016) 37:12193–202. doi: 10.1007/s13277-016-5079-x 27230679

