# Supplementary material for: An exploratory Case Report: integrated genomic and transcriptomic profiling of a spontaneous facial tumor in the endangered Milu deer (Elaphurus davidianus)
Source: Front Vet Sci. 2026 Oct 22;13:1933048. doi: 10.3389/fvets.2026.1933048 (PMC13735093; doi:10.3389/fvets.2026.1933048)
Supplement: Supplementary file 1 [file Table_2.docx]

Supplementary Table S2. Summary of RNA-seq quality control metrics

| Sample | Clean Reads (Million) | Q20(%) | Q30(%) | Error Rate (%) | % Mapped Reads | % Uniquely Mapped | % Multi-mapped | GC Content (%) | Adapter Contamination (%) | Mean Insert Size (bp) |
| --- | --- | --- | --- | --- | --- | --- | --- | --- | --- | --- |
| Control | 52.51 | 98.85 | 96.53 | 0.01 | 82.45 | 78.57 | 3.88 | 57.5 | 8.02 | 270.98 |
| Tumor | 47.72 | 98.70 | 96.15 | 0.01 | 88.60 | 83.97 | 4.63 | 58.9 | 0.20 | 263.03 |
